# A review of reusable insulin pens and features of TouStar—a new reusable pen with a dedicated cartridge

**DOI:** 10.1186/s13098-021-00763-z

**Published:** 2021-12-19

**Authors:** Robert Veasey, Carolin A. Ruf, Dmitri Bogatirsky, Jukka Westerbacka, Arnd Friedrichs, Mona Abdel-Tawab, Steffen Adler, Senthilnathan Mohanasundaram

**Affiliations:** 1 DCA Design International Ltd., Warwick, UK; 2 Sanofi-Aventis Deutschland GmbH, Frankfurt, Germany; 3 Sanofi, Paris, France; 4 Dr. Arnd Friedrichs Unternehmensberatung, Görlitz, Germany; 5 Zentrallaboratorium Deutscher Apotheker, Eschborn, Germany; 6 Sanofi, Mumbai, India

**Keywords:** Cartridge, Diabetes mellitus, Insulin pen, Glargine 300 U/mL

## Abstract

**Background:**

Since the introduction of the first reusable insulin pen, the advancement in the design of these pens is still ongoing to develop a safe, more efficacious, less painful, and easy to use insulin pen device.

**Main body:**

Possible errors in insulin delivery can occur at any stage of insulin delivery such as during the prescription stage, dispensing stage, or at administration stage. Mismatch of the insulin pen and cartridge is not uncommon and is a potential risk for individuals with diabetes due to serious consequences associated with incorrect insulin usage. The similarities in insulin cartridges of different manufacturers with regard to color and product names could lead to mix-up of insulin pens and cartridges. These unmet needs have led to the ongoing search for developing insulin pens that can address these errors and provide more efficacious and safer choices for patients with diabetes.

**Conclusion:**

This review provides an overview of currently available reusable pens in the market and highlights the features of TouStar^®^, a new reusable pen with a dedicated cartridge intended to mitigate the risk of mismatch of the cartridge.

## Insulin therapy and unmet need

The discovery of insulin molecule, a ground-breaking innovation in the history of diabetes care, has completed 100 years in 2021. In this time insulin therapy emerged as an indispensable tool in the arsenal of therapies used for diabetes management across the globe. However, errors in the insulin-related medication therapy can have serious consequences. The overdose of insulin can lead to severe hypoglycemia, causing seizures, coma, and even death; or under dosing of insulin may result in hyperglycemia and sometimes ketoacidosis [1].

In the past three decades, the use of insulin pens has received recognition as a promising insulin-delivery device from healthcare providers as well as patients due to advantages such as less pain, ease of application and benefits in achieving glycemic control. The superiority of insulin pens has been established over conventional use of vials and syringes, particularly in providing more accurate and safer dose delivery, comfort and convenience of use, and lower risk of errors in the administration process [2–5]. The continual improvement in the technology of pens has contributed to the improved adherence to insulin therapy and quality of life, increased patient preference for insulin pens, and decreased overall healthcare cost [2–8]. Despite the rapid advancement in the design of insulin pens to provide safe, more efficacious, less painful, and easy to use insulin pen device, the currently available pens in the market have further scope of improvement [9].

Though insulin errors can occur at any of the steps of the medication use process, including prescribing, transcribing, dispensing, and administration, the majority of these errors occur during administration [10]. Mix-up of the insulin pens/cartridges such as choosing the wrong pen/cartridge when using both long- and short-acting insulin is not uncommon and is a potential risk for individuals with diabetes due to adverse consequences associated with incorrect insulin usage [11]. The similarities in insulin cartridges of different manufacturers with regard to color and product names could lead to this mix-up of insulin pens and cartridges. In addition, mistaking a large dose of rapid-acting insulin for long-acting insulin can be potentially fatal [11]. The color differentiation of insulin pens has been able to eliminate this risk partially. In particular, color differentiation features work better for disposable insulin pens, where prominent surfaces of the external components can have different colors for fast- or long-acting insulins. However, when using reusable insulin pens, cartridge differentiation is critical and even more difficult.

These unmet needs have led to the ongoing search for developing better insulin pens that can address these errors and provide more efficacious and safer insulin pens. An insulin pen with a dedicated cartridge, wherein cartridge is pre-assembled into the labelled cartridge holder and will be replaced as a combined unit, could help to overcome the issue of mix-up of insulin pens and cartridges. The purpose of this review is to provide an overview of currently available reusable pens in the Indian market and to highlight the features of TouStar^®^ pen (delivering insulin glargine 300 U/mL), a new reusable pen with a dedicated cartridge, launched in India.

## Overview of reusable pens

Among the various advantages of reusable pens, the most important benefits include durability that aids in removing a barrier of large storage space required for disposable insulin pens, and flexibility in carrying 3–5-day supply [12]. Meanwhile, the reusable insulin pens are more accurate in terms of fulfilling the dose accuracy according to international standards (ISO-11608-1) and equipped with additional features such as audible, visual and tactile feedback during dose dialing to support easy application and may reduce the chances of human errors during handling [13–15]. In addition, the newer reusable insulin pens provide a key benefit of correcting a selected dose without losing insulin as compared to older pens wherein it was not possible to dial “back” the dose due to one directional dial restrictions. In older reusable pens, the steps to reset the pen when changing to a new cartridge were quite difficult, wherein the piston rod needs to be re-wound to its starting point. However, the advancement in the newer reusable pens allows the user to just push the piston rod back into the pen instead of a rotating step.

In addition to the afore-mentioned advantages, in various settings reusable pens may be cost-effective as compared to vials and syringes [16]. Further, these insulin delivery pens are environmentally friendly that could provide ecological advantage in terms of contributing to less plastic waste, compared to disposable pens.

An assessment of reusable insulin pen devices among patients with diabetes in developing countries reported that the participants ranked AllStar^®^ highest on majority (75%) of the key features (easiest to use overall, 52%; easiest to read the dose, 42%; and easiest to self-inject, 39%) compared to other reusable pens [17]. Currently there are several reusable pens available in the global market with a wide range of advantages that enhance the dose accuracy and help to alleviate user or medication errors, thereby increasing the efficiency of insulin therapy [9]. Table 1 summarizes the details of the currently available reusable insulin pens in the market [18–25].

**Table 1:** Features of TouStar and other reusable pens

Feature		TouStar	AllStar	NovoPen 4	HumaPen Ergo II	INSUPen Pro
Insulin type		Toujeo^®^ U300	Any Sanofi U-100 (Lantus^®^, Insulin lispro, Apidra^®^)	Penfill^®^	Huminsulin^®^ or Humalog^®^	Insugen-R Refil, Insugen-N Refil, Insugen-30/70^®^ Refil, Insugen-50/50 Refil and Basalog Refill
Maximum dose that can be delivered in one injection (increment in units)		80 U (1 U)	80 U (1 U)	60 U (1 U)	60 U (1 U)	60 U (1 U)
Total capacity		450 U	300 U	300 U	300 U	300 U
User can *reverse dial* without losing insulin		Yes	Yes	Yes	Yes	Yes
"End of cartridge" dial limit when dialing doses		Yes	Yes	Yes	No	No
Simple "push-to-reset" plunger (no screwing required)		Yes	Yes	Yes	Yes	Yes
Readability, high contrast, magnified dose numbers	Character height (mm) after magnification, if applicable (SD)	3.3 (0.1)	3.2 (0.1)	3.4 (0.0)	3.1 (0.1)	3.0 (0.0)
	Character width (mm) after magnification, if applicable (SD)	2.1 (0.0)	2.1 (0.0)	2.4 (0.0)	1.9 (0.0)	2.0 (0.0)
Compactness	Length with Cap fitted in mm (SD)	154.6^b^ (0.2)	154.6^b^ (0.2)	157.1 (0.0)	165.2 (0.1)	166.6 (0.1)
	Diameter at widest point excluding pocket clip in mm (SD)	16.3^b^ (0.0)	16.3^b^ (0.0)	15.9 (0.0)	20.2 (0.0)	17.4 (0.0)
Weight—total mass in g, including a full cartridge (SD)		25.9^a^ (0.0)	28.5 (0.0)	56.3 (0.1)	46.6 (0.1)	38.1 (0.0)
Dial extension measured at 60 U from end of dose button to housing in mm. (SD)		27.5^b^ (0.1)	27.5^b^ (0.1)	27.9 (0.0)	44.2 (0.1)	44.2 (0.1)

Differences were tested using a two-sided t-test at a significance level p = 0.001

*SD* standard deviation

^a^Statistically significant difference between TouStar and all other pens

^b^Statistically significant difference between TouStar/AllStar and remaining pens

## Factors determining choice of insulin pen

Given that the insulin pens are associated with less needle-phobia and the ease of injecting insulin contributes to increased patient compliance with therapy, the selection of an appropriate insulin administration device according to patients’ preference is important to improve adherence and to maintain persistence with basal insulin therapy [26, 27].

Multiple factors are attributed in determining the most appropriate insulin pen among the available options in the market which include clinical and patient-related factors.

### Biomedical factors

- The advantage of the dose accuracy of insulin pens compared to insulin delivery using syringes is one of the important factors considered while choosing an application device.
- Length of needle is a crucial factor in the selection of insulin pen because generally all kinds of patients, irrespective of their age, have needle phobia owing to the perception of more pain associated with a higher length of the needle [28].
- Resolution of dose increment: the required resolution of dose increment varies individually depending on each patient’s condition. For example, children with type 1 diabetes, persons with brittle diabetes, and those with very low dose requirements of insulin need a pen device that will deliver insulin in smaller increments such as 0.5 units. Thus, patients can opt to use insulin pens that provide the desired resolution of dose increment depending on their condition.
- Suitability for users with different needs, such as dexterity-challenged persons: insulin pens with a legible display of dose, audible, visual and tactile feedback after each dialed dose, and plunger buttons that need low injection force as well as short dial extensions when dialing and injecting higher doses, are required for different user groups, such as patients with poor dexterity or elderly users [26, 29–31].

### Psychosocial factors

In patient-centric factors, the attractiveness of pen substantially contributes as a deciding factor in the choice of insulin pen. The size, color, and discreteness of pen design are factors that may influence patient preference. Other factors like comfort in using the device and ease of transport may also contribute as a deciding factor for many patients in determining best suitable insulin pen that can have minimal impact on patient’s quality of life [29, 32].

### Pragmatic factors

- Cost of insulin refills and needles along with the device are also vital contributors that need to be considered by healthcare professionals, especially for patients with low socioeconomic status.
- Expected duration of insulin therapy required for each patient is another factor that can influence the choice of insulin device. In general, for patients requiring long-term insulin therapy, reusable insulin pens may be preferred; while for those who require insulin therapy for a short duration, disposable insulin pens are preferred [29].
- For patients needing intensification or de-escalation of insulin regimen in near future, the compatibility of insulin device with multiple insulin preparations is an important consideration.
- Familiarity of the pen further benefits patients in terms of well-versed knowledge of handling of the device that could reduce the risk for handling and dosing errors.
- Availability of insulin cartridges that are compatible to the prescribed insulin pen and ancillary supplies are other factors that are important for patients living in rural areas where easy access to medicine is limited.
- Monthly dose requirement of insulin and capacity of the device are also important factors in determining the best suitable insulin pen [29].

### Medication counseling factors

Initial and regular counselling of patients to educate about proper technique of using insulin pens is recommended and is an important factor in achieving better glycemic control and improved medication adherence [33]. Generally, healthcare professionals (HCPs) play an important role in counselling patients about the use of insulin pens and the risk of error in dose delivery. Due to heavy patient load in routine clinical practice, the time taken to teach and their ability to provide post-counselling follow-up may also be considered important in influencing the choice of insulin pen. Similarly, from patients’ point of view, time taken to learn is an important aspect that determines their preference for any insulin pen. The insulin pens that are easier to use and learn are preferred by patients [29].

## Overview of TouStar features and comparison with other available reusable pens

**Fig. 1:**
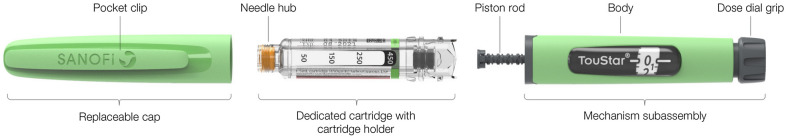
Parts of TouStar

TouStar pen is an advanced version of AllStar pen and is intended to be used in conjunction with the insulin glargine 300 U/mL in a dedicated cartridge (Toujeo^®^ 1.5 mL cartridges) to deliver insulin through subcutaneous injection using commercially available needles. The key technical features of TouStar pen are summarized in Table 1, and Fig. 1 presents the design of the pen device.

The dedicated cartridge comprises a Toujeo cartridge pre-assembled into the labelled cartridge holder and will be replaced as a combined unit. The dedicated joint interface between the cartridge holder and pen body is intended to prevent users from using other insulin cartridges and accidentally using a prandial insulin or insulin of other concentration with this pen to mitigate potentially adverse dosing errors. Thus, this pen provides a facility of using a dedicated cartridge that helps in avoiding the mismatch error of the insulin pen and insulin refills and therefore can help to eliminate the risk of administering incorrect insulin and associated serious complications. Among the currently available reusable insulin pen devices in the market, there is no pen device that provides this feature of dedicated cartridges.

The cartridge holder allows visibility of the deliverable volume before and during the use of the pen injector. A scale on the cartridge holder label helps to determine the residual deliverable volume in the cartridge. A large-print dose window is provided for better visibility of the selected dose values.

**Fig. 2:**
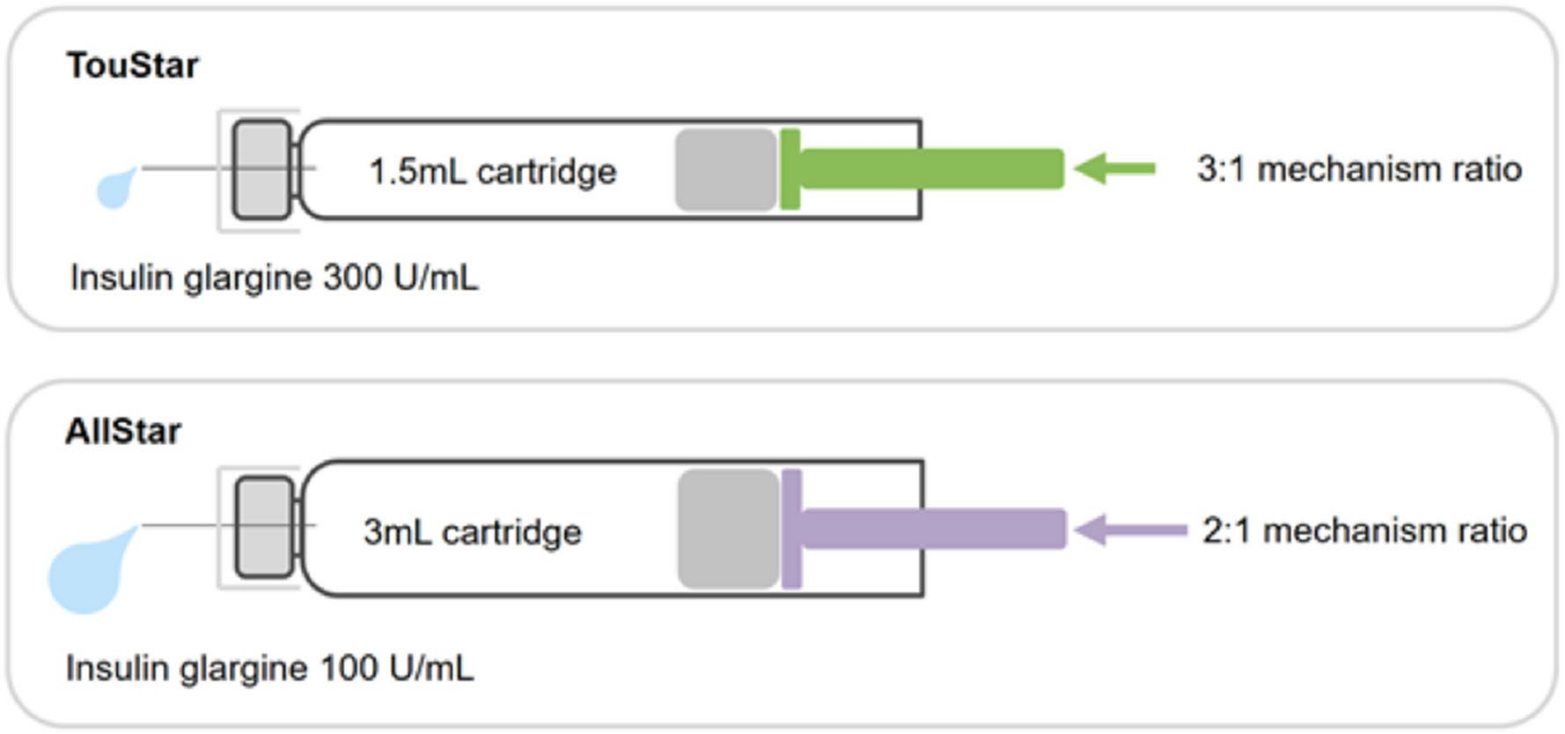
An illustration of the mechanism ratio and volume of insulin delivered by TouStar and AllStar for an equivalent dose of insulin. Mechanism ratio: the ratio of movement of the dose button relative to the piston rod when delivering a dose

Owing to the higher amount of insulin per unit volume and size of the cartridge used, the TouStar pen contains a higher-ratio mechanism that was designed to make smoother dose delivery and easier than previous versions of the AllStar pen with low injection force (Fig. 2) [34]. At the same time, TouStar retains the same dial extension as AllStar. Small dial extension of pen may make it easy to use and convenient for patients with poor dexterity. A low injection force facilitates a simpler operation and reduced injection-site pain [35, 36].

This device provides selection of range of doses up to 80 U in increments of 1 U. The wide deliverable dose limit of 80 U at a time avoids the need for re-dialing or injecting twice for administration of large doses. The dose delivery in 1 U increment is common among several reusable pens available in the market; whereas few reusable pens, such as HumaPen^®^ Luxura™ HD have the ability to deliver insulin in half-unit increments [18, 37, 38].

The total deliverable dose in the TouStar cartridge is 450 U which may allow patients to use this insulin cartridge for a long duration. All the other available products have a total deliverable capacity of 300 U or less [18–25]. Mechanical last dose stop feature of TouStar helps to prevent dialed dose from exceeding the remaining content thereby reducing the risk of underdosing. This feature of TouStar is in accordance with the AllStar pen device and with other reusable pens in the market as well [24].

Dose dial grip extension of ≤ 35 mm provides short dial-out distance. This feature may be useful for patients with different hand sizes (such as female or elderly patients with low finger strength, with small hands). Tactile, visual, and audible feedback at each dose-setting helps patients to confirm the desired dosage delivery.

In addition, it is possible to inject multiple doses with a penalty-free dose correction facility, thus allowing to correct an over-dialed dose with a needle fitted without wasting the insulin dose. The device will provide its total deliverable volume after the correction of an 80 U dose.

The TouStar pen could be used for three years and is similar to most of the available reusable pens (HumaPen Luxura HD, NovoPen^®^ 3, INSUPen). Only for NovoPen^®^ 4, the in-use lifetime is five years when the pen is used for three daily injections.

TouStar has a dose-dial grip hold time of 5 s for full dose delivery. It enables delivery of volume within safety margin when the injection is made without holding time. This feature is observed in few marketed pen devices (HumaPen Luxura HD), while other devices require a hold time of 10 s to deliver a full dose of the drug (AllStar, INSUPen) [18, 24, 25].

## Assessment of features of TouStar and four other reusable insulin pens available in India

An assessment was conducted to evaluate the relevant pen dimensions, weight and the font size within the dose window of five representative reusable insulin pens. The assessment parameters include: weight of the pen with insulin cartridge and cap attached, length of the extended dose dial extension when the pen is dialed to 60 U, length of the pen dialed to the maximal number of units with cap attached, length of the pen with cap fitted, maximum diameter of pen body, number height and number width within dose window. The measurements were carried out in an external laboratory (Dr. Arnd Friedrichs Unternehmensberatung, Görlitz, Germany) in February 2021 under standardized conditions. Five pens of each model were used. Each measurement was repeated in triplicate. The length and maximum diameter were measured with a pair of calibrated electronic calipers. For the measurement of the pen weight, a calibrated precision balance was used (OHAUS PA2101). The size of the number within the dose window was measured with a precision magnifying lens with integrated scale and a seven-fold magnification. All parameters were assessed without attaching a needle to the pen. Table 1 summarizes the observations from this assessment.

The pen characteristics investigated in this assessment may have impact on the confidence of users and ease of pen use. Improved ease of use may result in convenience of treatment and improved treatment compliance. A lower pen weight may be convenient for everyday transportation, ease of operation and may be particularly important for elderly patients with neuromuscular impairments or hand fatigue. Smaller pen dimensions and a shorter dial extension may facilitate easier operation, particularly for patients with small hands and limited dexterity. A larger dose digit facilitates easier dose setting because of increased readability. The selection of pen should be individualized according to the needs of the patient.

## Future scope of further research

Further studies assessing the injection force of reusable pens are needed. Furthermore, clinical experience data, real-world evidence, and studies evaluating user preference of the reusable pens available in India will help to establish a concrete evidence with respect to its appropriate use in routine clinical practice.

## Conclusion

The unique design features make TouStar pen the first reusable insulin pen with a dedicated cartridge for long-acting insulin glargine 300 U/mL to deliver the appropriate dose intended to mitigate the risk of mismatch of the insulin glargine 300 U/mL cartridge with another insulin cartridge or the wrong insulin pen. Future real-world studies are necessary to understand the user experience with reusable pens in routine clinical practice.

## Data Availability

Data sharing is not applicable to this article as no datasets were generated or analyzed during the current study.

## Abbreviation

HCPs: Healthcare professionals
